# Evaluation of Additional Treatment for Residual Cases After Endoscopic Papillectomy of Duodenal Ampullary Tumors

**DOI:** 10.1002/deo2.70374

**Published:** 2026-07-01

**Authors:** Yusuke Kurita, Keiki Nagai, Noriki Kasuga, Kensuke Kubota, Yuji Fujita, Yusuke Sekino, Hiroki Uechi, Yuji Koyama, Shintaro Tsujikawa, Shota Matsumoto, Shigeki Tamura, Yu Honda, Shin Yagi, Takeshi Iizuka, Sho Hasegawa, Takamitsu Sato, Kunihiro Hosono, Noritoshi Kobayashi, Yuki Honma, Ryusei Matsuyama, Jotaro Harada, Masato Yoneda, Satoshi Fujii, Itaru Endo, Atsushi Nakajima

**Affiliations:** ^1^ Department of Gastroenterology and Hepatology Yokohama City University School of Medicine Yokohama Japan; ^2^ Department of Hepato‐Biliary‐Pancreatic Medicine NTT Medical Center Tokyo Tokyo Japan; ^3^ Department of Gastroenterology Yokohama Rosai Hospital Yokohama Japan; ^4^ Department of Oncology Yokohama City University School of Medicine Yokohama Japan; ^5^ Department of Gastroenterological Surgery Yokohama City University School of Medicine Yokohama Japan; ^6^ Department of Molecular Pathology Yokohama City University School of Medicine Yokohama Japan

**Keywords:** adenocarcinoma, ampullary carcinoma, ampullary tumor, endoscopic papillectomy, pancreatoduodenectomy

## Abstract

**Background and Aims:**

This study aimed to assess the prognosis and outcomes of additional treatment for residual adenocarcinoma and adenoma following endoscopic papillectomy (EP) for ampullary tumors.

**Methods:**

Of 191 patients who underwent EP, 43 with adenocarcinoma or adenoma and either positive or unknown pathological margins were included. Cases with positive or unknown pathological margins and residuals on endoscopic observation after EP were defined as endoscopic residuals, and cases without endoscopic residuals were defined as pathological residuals. The outcomes between these groups were compared to evaluate additional treatment.

**Results:**

A total of 12 cases of adenocarcinoma were identified, with pathological residuals in 10 cases and endoscopic residuals in two cases. Of the two cases with endoscopic residuals, one underwent additional surgical resection, while the other was observed and subsequently showed disease progression. Among the 10 cases with pathological residual adenocarcinoma, four underwent additional surgery, but no cancer was detected in the resected specimens. The remaining six cases showed no disease progression during the observation period, suggesting that pathological residual adenocarcinoma may have been completely eradicated by EP. Residual adenoma was detected in 31 cases, with pathological residuals in 20 cases and endoscopic residuals in 11 cases. Adenoma recurrence occurred in three cases, all of which were successfully managed with endoscopic treatment.

**Conclusions:**

If the pathological margin is positive or unknown, additional treatment is necessary for cases with endoscopic residuals after EP. However, in the absence of endoscopic residuals, follow‐up observation may be carefully considered in selected cases with caution.

**Trial Registration:**

N/A.

## 1 Introduction

Endoscopic papillectomy (EP) is an established treatment for ampullary neoplasms. Recent studies have also demonstrated its utility in early‐stage duodenal papilla cancer [1, 2]. However, complete resection rates following EP range from 47%–81% [1, 2, 3, 4, 5], and residual tumors are frequently encountered. Managing these residual lesions remains a significant clinical challenge. Reports have highlighted the efficacy of additional endoscopic [4, 6, 7] and surgical treatments [2, 8, 9, 10] for residual or recurrent cases. While additional surgical resection is considered beneficial in cases with confirmed vascular invasion or invasive carcinoma, the optimal approach for residual disease remains undefined. No consensus exists on selecting the most appropriate intervention, particularly in differentiating treatment strategies for pathological residuals (positive or unknown pathological margins) and endoscopic residuals (visible residual lesions on post‐EP endoscopy).

This study aimed to evaluate the outcomes and prognosis of additional treatment for residual adenocarcinoma and adenoma following EP for duodenal ampullary tumors.

## 2 Methods

**FIGURE 1:**
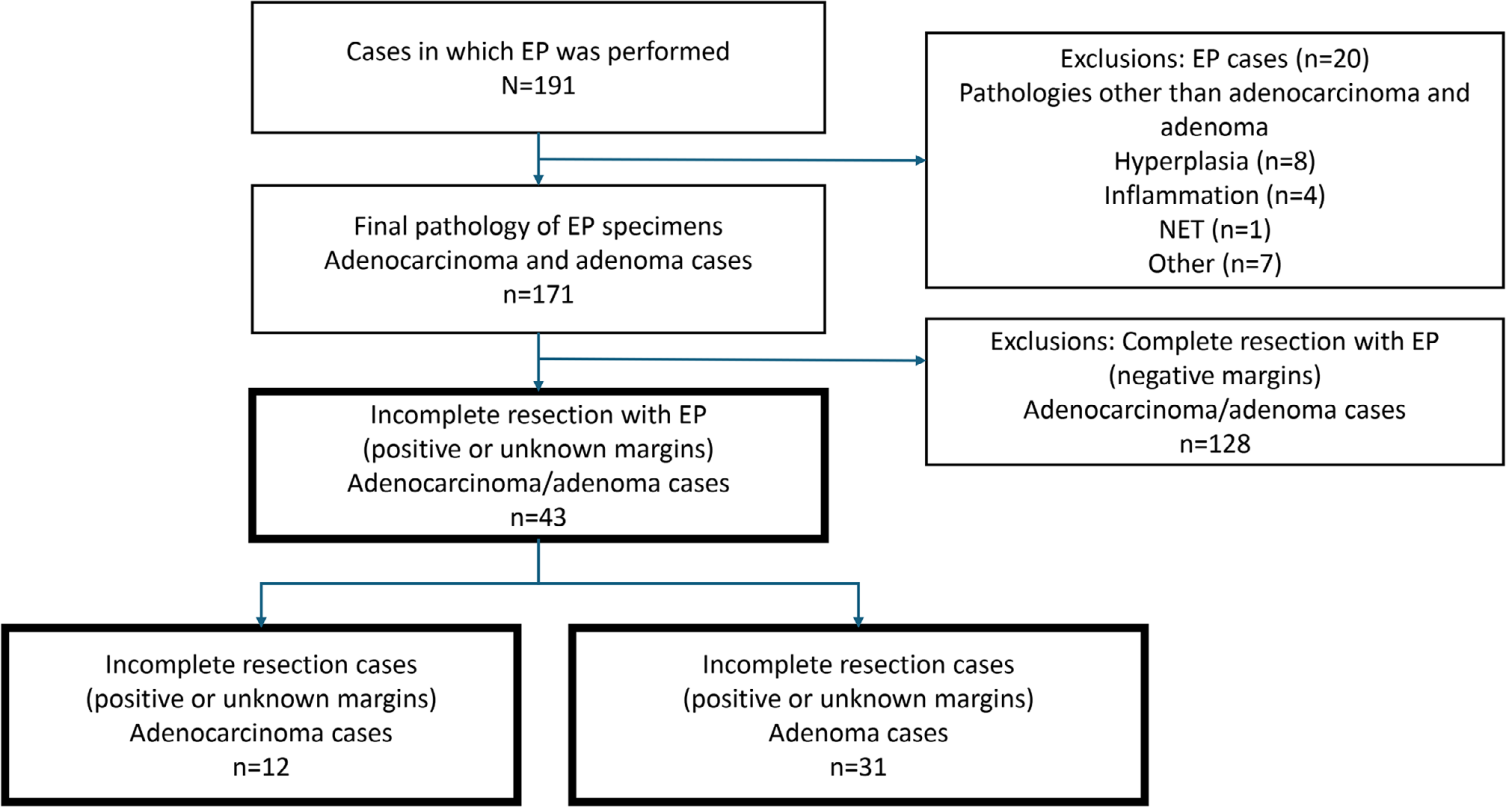
Flowchart for the selection of patients with positive or unknown margins.

This multicenter retrospective study included patients who underwent EP between 2005 and 2024 at three institutions. Only cases with a final diagnosis of adenocarcinoma or adenoma were analyzed. Cases with complete resection (negative margins, no vascular invasion) were excluded, while those with incomplete resection (positive or unknown margins) were included. Inclusion criteria are shown in Figure 1.

The study was approved by the IRB of Yokohama City University Hospital (F241200003). As a retrospective study with no patient contact, informed consent was obtained through an opt‐out process. Patients who declined were excluded.

### 2.1 Indication and Procedure of EP

Indications for EP at the three institutions included: (1) endoscopic confirmation of neoplastic lesions, (2) histopathological evidence of neoplasms in biopsy specimens, (3) absence of intraductal extension into the pancreatic or bile ducts as confirmed via endoscopic ultrasound (EUS) or intraductal ultrasound, (4) no evidence of advanced malignancy, including ulcerative lesions or severe duodenal deformities, and (5) absence of regional lymph node involvement, ascites, or distant metastases on imaging studies. In cases where the preoperative biopsy diagnosis was adenocarcinoma, only lesions that appeared endoscopically confined to the mucosal layer were considered eligible for EP.

A single‐channel duodenoscope (JF‐260 V, TJF‐260 V, or TJF‐Q290V; Olympus, Tokyo, Japan) was used for EP. Resection was performed using a snare (Rotatable Polypectomy Snare 13 or 20 mm, MA, Boston Scientific, Marlborough, MA, USA), targeting the area from the upper part of the frenulum to the tip of the hooding fold. The electrosurgical unit was set to the end‐cut mode, and the tumor was resected using ICC200 (ERBE Elektromedizin, Tübingen, Germany) and VIO300D (ERBE Elektromedizin, Tokyo, Japan). The output settings for EP with ICC200 included end‐cut mode, effect 3 (output limit 120 W), and soft coagulation (output limit 30 W). EP was conducted with the VIO300D in Endo Cut I mode (Effect 2, Cut duration 2, Cut interval 3).

Complete en bloc resection was performed when the lesion was entirely resected. However, for larger lesions or cases where gross tumor tissue remained despite an attempted en bloc resection, piecemeal resection was carried out in multiple sessions. In select cases, preventive closure of the frenulum was achieved using clips [11]. If intraoperative bleeding occurred, endoscopic hemostasis was performed at the discretion of the endoscopist, utilizing argon plasma coagulation (APC), hypertonic saline epinephrine solution injection, or clipping. To reduce the risk of post‐procedural adverse events, 5‐French (Fr) pancreatic stents were placed to prevent post‐endoscopic pancreatitis [12], and 7‐Fr biliary stents were implanted to reduce the risk of post‐EP cholangitis.

### 2.2 Pathological Assessment

Endoscopically resected specimens were collected for histological evaluation, with tumor size determined using endoscopic measurement prior to formalin fixation. In cases of piecemeal resection, tumor size was estimated based on the endoscopic visual field. The specimens were then assessed by an experienced pathologist.

The final pathological diagnosis was determined using the Union for International Cancer Control TNM Classification of Malignant Tumors, eighth Edition [13]. Based on the depth of cancer invasion, carcinomas were classified as follows: Tis for carcinoma in situ; T1a for tumors confined to the ampulla of Vater or sphincter of Oddi; T1b for tumors invading beyond the sphincter of Oddi and/or extending into the duodenal submucosa; T2 for tumor invasion into the muscularis propria of the duodenum; T3 for tumors with direct invasion into the pancreas or peripancreatic tissue; and T4 for tumors infiltrating the celiac axis, superior mesenteric artery, or common hepatic artery.

In this study, tumors confined to the mucosa were subclassified as T1a(M), whereas lesions involving the sphincter of Oddi were subclassified as T1a(OD), based on the General Rules for Clinical and Pathological Studies on Cancer of the Biliary Tract, as edited by the Japanese Society of Hepato‐Biliary‐Pancreatic Surgery. Adenocarcinomas were categorized based on their differentiation into well‐differentiated (tub1), moderately differentiated (tub2), or poorly differentiated (por) subtypes.

Resection margins were evaluated both horizontally and vertically, using the following criteria: x (unknown), where margin status could not be assessed; − (negative), indicating no cancer involvement at the margin; and + (positive), signifying tumor involvement at the margin. Lymphovascular invasion was defined as the presence of endolymphatic vessel infiltration or microvascular emboli.

### 2.3 Follow‐up After EP

Close observation was conducted at 4 weeks, 6 months, and 1 year following EP, followed by annual evaluations for 5 years. Follow‐up included clinical examination, duodenoscopy, and EUS. If endoscopic surveillance detected residual or recurrent tumors, a biopsy was performed to confirm the pathology. For cases where the final pathology confirmed adenocarcinoma, additional follow‐up was supplemented with computed tomography (CT).

Follow‐up data for patients who underwent surgery were obtained through a review of surgical and oncologic records.

### 2.4 Definition of EP Residual Cases

In this study, residual cases were defined as those with positive or unknown pathological margins. Cases in which adenomas or adenocarcinomas were detected at the initial endoscopic follow‐up and confirmed by biopsy, were classified as endoscopic residual cases. Among cases of incomplete resection with positive or unknown margins, those without endoscopic residual disease were categorized as pathological residual disease cases. Recurrence was defined as tumor presence during the second or subsequent endoscopic follow‐up or when CT or other imaging modalities detected tumors in other organs.

### 2.5 Additional Treatment for Residual Case After EP

Pancreatoduodenectomy was recommended for adenocarcinoma with positive or unknown margins after EP. Surgery was also advised for tumors localized to the sphincter of Oddi, cases with invasion deeper than T1a(OD), or instances where endoscopic follow‐up revealed residual tissue, regardless of resection margin status, ductal invasion, or lymphatic involvement. Patients deemed high‐risk for surgery due to poor general condition, or those who declined surgical resection, were managed with close follow‐up.

For incompletely resected adenomas, additional endoscopic treatment was performed. The choice of modality was at the endoscopist's discretion, including repeat EP (re‐EP), APC, or radiofrequency ablation (RFA). In select cases, surgical resection was performed based on the endoscopist's judgment.

### 2.6 Endpoints

The primary endpoint was to evaluate the outcomes and prognosis of additional treatment for pathological and endoscopic residual adenocarcinoma. Prognosis was assessed based on primary tumor progression, recurrence, and patient survival. Additionally, we evaluated the feasibility of achieving curative resection for adenocarcinoma via EP. The secondary endpoint was to evaluate the outcomes and prognosis of additional treatment for pathological and endoscopic residual adenoma.

Cases undergoing additional surgical resection were also analyzed for surgical outcomes and adverse events.

### 2.7 Definition of Curative Resection of EP

Curative resection of EP for adenocarcinoma was defined as cases where no residual cancer was detected in additional surgical resection or where no recurrence, tumor progression, or metastatic spread was observed after at least 1 year of follow‐up. For adenoma, curative resection was defined as cases in which no residual adenoma or malignant transformation (adenocarcinoma) was identified after additional surgical resection or in which no recurrence, progression of the primary lesion, or metastatic development occurred during at least 1 year of follow‐up.

Cases that developed recurrence during follow‐up, including recurrence after 1 year of follow‐up, were not considered curative resections.

Cases requiring additional endoscopic treatment, including re‐EPs, APC, or RFA, were considered non‐curative.

Cases lacking at least 1 year of follow‐up data after EP were excluded from the analysis of curative resection outcomes.

### 2.8 Statistical Analysis

All statistical analyses were performed using SPSS version 27.0 (IBM Corp., Armonk, NY, USA). To assess associations between variables, univariate analysis was performed using the Mann–Whitney U test for continuous variables and Pearson's chi‐squared test or Fisher's exact test for categorical data.

## 3 Results

### 3.1 Patients’ Characteristics

EP was performed in 191 cases, with 171 diagnosed as adenocarcinoma or adenoma. Of these, 43 had positive or unknown margins (12 adenocarcinoma and 31 adenoma). Figure 1 shows the selection flow; Table 1 summarizes patient backgrounds.

**TABLE 1:** Characteristics of patients with pathological residual disease following endoscopic papillectomy (EP) (*n* = 43).

	Adenocarcinoma	Adenoma	
	(*n* = 12)	(*n* = 31)	
Age, median (range), years	74 (53–88)	66 (38–83)	0.081
Sex, male (%)	7 (58.3)	18 (58.1)	0.987
Familial adenomatous polyposis (%)	1 (8.3)	3 (9.7)	1.000
Tumor size, median (range), mm	15 (8–25)	12 (5–20)	0.002
Procedure time for EP, median (range), mm	53.5 (21–140)	37.0 (15–101)	0.095
Biliary stenting of EP (%)	11 (91.7)	29 (93.5)	1.000
Pancreatic stenting of EP (%)	12 (100)	30 (96.8)	1.000
Mode of resection of EP (%)			
En bloc resection	11 (91.7)	26 (83.9)	0.659
Piecemeal resection	1 (8.3)	5 (16.1)	
Endoscopic residual case (%)	2 (16.7)	11 (35.5)	0.280
Additional treatment (%)			
Surgical resection	5 (41.7)	2 (6.5)	0.004
Endoscopic treatment	0	11 (35.5)	
Observation	7 (58.3)	18 (58.1)	

Abbreviation: EP, endoscopic papillectomy.

### 3.2 Results of EP, Additional Treatment, and Prognosis in Adenocarcinoma

Residual disease was observed in 12 cases of adenocarcinoma with positive or unknown pathological margins (Table 2). Among these, two cases exhibited endoscopic residual disease, confirmed through endoscopic observation after EP, while 10 cases had pathological residual disease detected on histological evaluation.

**TABLE 2:** Results of endoscopic papillectomy (EP), additional treatment, and prognosis after follow‐up in adenocarcinoma cases.

Adenocarcinoma total (*n* = 12)	Pathological residual case (*n* = 10)	Endoscopic residual case (*n* = 2)	*p*
Tumor size, median (range), mm in EP	17 (10–21)	14.0 (12–16)	0.364
Mode of resection in EP (%)			1.000
En bloc/Piecemeal	9 (90.0)/1 (10.0)	2 (100)/0 (0)	
Final pathological diagnosis of EP			0.598
Tis/T1a(M)/T1a(OD)	6/2/2	1/0/1	
Histological grade (%)			0.318
Tub1/Tub2	9 (90.0)/1 (10.0)	1 (50.0)/1 (50.0)	
Histological subtype (%)			0.318
Intestinal/Pancreatobiliary	9 (90.0)/1 (10.0)	1 (50.0)/1 (50.0)	
Lymphovascular invasion (%)	0 (0)	0 (0)	NA
Resected margin of EP (‐)/(+)/(x)	0/5/5	0/1/1	1.000
Horizontal (‐)/(+)/(x)	8/1/1	1/1/0	0.368
Vertical (‐)/(+)/(x)	2/4/4	1/0/1	0.487
Additional surgical resection (%)	4 (40.0)	1 (50.0)	1.000
Adenocarcinoma in additional surgical specimens (%)			
Absence	4 (40.0)	−	0.200
Presence	0 (0)	1 (50.0)	
Additional endoscopic treatment (%)	0 (0)	0 (0)	NA
Observation without additional treatment (%)	6 (60.0)	1 (50.0)	1.000
Follow‐up period after EP, months, mean (range)	56.2 (13.6–86.6)	29.6 (21.0–38.2)	0.485
Growth of primary tumor or recurrence (%)	0 (0)	1 (50.0)	0.167
Status of clinical follow‐up (%) Death	0 (0)	0 (0)	NA
Possibility of curative resection via EP (%)	100 % (10/10)	0% (0/2)	0.015

Abbreviations: EP, endoscopic papillectomy; Tis, carcinoma in situ; T1a(M), tumors confined to the mucosa; T1a(OD), lesions involving the sphincter of Oddi; Tub1, well‐differentiated; Tub2, moderately differentiated; (‐), no cancer involvement at the margin; (+), tumor involvement at the margin; (x), margin status could not be assessed.

**FIGURE 2:**
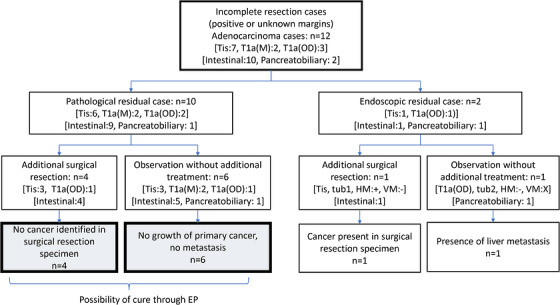
Clinical course of adenocarcinoma with positive or unknown endoscopic papillectomy (EP) margin. Among 12 cases of residual adenocarcinoma with positive or unknown pathological margins, two were identified as endoscopic residual cases, where adenocarcinoma was detected through endoscopic observation and biopsy following EP. The remaining 10 cases were classified as pathological residual cases, in which no visible tumor was detected during follow‐up endoscopy. All 10 pathological residual cases exhibited no evidence of recurrence, suggesting that curative resection was achieved through EP. EP, endoscopic papillectomy; Tis, carcinoma in situ; T1a(M), tumors confined to the mucosa; T1a(OD), lesions involving the sphincter of Oddi; HM, VM.

Although the sample size was small, there was no significant difference in resection outcomes between pathological residual cases (*n* = 10) and endoscopic residual cases (*n* = 2). The mean follow‐up period after EP was 56.2 months (13.6–86.6) for pathological residual cases and 29.6 months (21.0–38.2) for endoscopic residual cases. Among the 10 cases of pathological residual disease, four patients underwent additional surgical resection, but no residual cancer was identified in the surgical specimens. The remaining six cases were managed with follow‐up observation, and none showed tumor progression or recurrence. In one of the two cases of endoscopic residual disease, additional surgical resection confirmed the presence of residual cancer in the resected specimen. The second case was initially managed with surveillance, but tumor progression and subsequent liver metastasis were observed. In both endoscopic residual cases, curative resection was not achieved by EP (Figure 2). The clinical course for cases with additional treatment, cases, and observation cases was shown in Table S1.

The clinical course for cases with pathological positive and unknown margins is shown in Table S2. Cases showing positive/unknown or negative vertical margins are shown in Table S3, while cases where positive/unknown or negative horizontal margins are shown in Table S4.

### 3.3 Results of EP, Additional Treatment, and Prognosis After Follow‐up in Adenoma

Among 31 adenoma cases with positive or unknown margins, 11 had endoscopic residual disease, and 20 had pathological residuals (Table 3). No significant differences were observed in the clinical characteristics or EP resection outcomes between the pathological residual and endoscopic residual groups.

**TABLE 3:** Results of endoscopic papillectomy (EP), additional treatment, and prognosis after follow‐up in adenoma cases.

Adenoma Total (*n* = 31)	Pathological residual case (*n* = 20)	Endoscopic residual case (*n* = 11)	*p*
Tumor size (mm), median (range), in EP	12 (5–20)	12 (5–20)	1.000
Mode of resection of EP (%)			
En bloc	18 (90.0)	8 (72.7)	0.317
Piecemeal	2 (10.0)	3 (27.3)	
Resected margin of EP (‐)/(+)/(x)	0/9/11	0/3/8	0.452
Horizontal (‐)/(+)/(x)	8/2/10	2/1/8	0.431
Vertical (‐)/(+)/(x)	1/9/10	2/2/7	0.228
Additional surgical resection (%)	0 (0)	2 (18.2)	0.118
Adenoma in additional surgical specimens (%)			
Absence	−	0 (0)	NA
Presence	−	2 (18.2)	
Additional endoscopic treatment (%)	2 (10.0)	9 (81.8)	<0.001
Re‐EP	0 (0)	5 (45.5)	
APC	1 (5.0)	3 (27.3)	
RFA	1 (5.0)	0 (0)	
Details of treatment unknown	0 (0)	1 (9.1)	
Observation without additional treatment (%)	18 (90.0)	0 (0)	<0.001
Follow‐up period after EP, months, mean (range)	41.5 (0.7–128.9)	50.4 (3.4–151.9)	0.502
Growth of primary tumor or recurrence (%)	2 (10.0)	1 (9.1)	1.000
Status of clinical follow‐up (%)			
Death due to a duodenal ampullary tumor	0 (0)	0 (0)	NA
Death due to other causes	2 (10.0)	0 (0)	0.527
Possibility of curative resection via EP (%)	75.0 % (12/16)	0 % (0/11)	<0.001

Abbreviations: APC, argon plasma coagulation; EP, endoscopic papillectomy; Re‐EP, repeat endoscopic papillectomy; RFA, radiofrequency ablation; (‐), no cancer involvement at the margin; (+), tumor involvement at the margin; (x), margin status could not be assessed.

Additional surgical resection was performed in two cases of endoscopic residual disease, and adenomatous lesions were confirmed in both surgical specimens. In each case, the attending physician at the time deemed additional treatment necessary based on the clinical findings. Additional endoscopic treatment was administered in two cases of pathological residual disease and nine cases of endoscopic residual disease.

**FIGURE 3:**
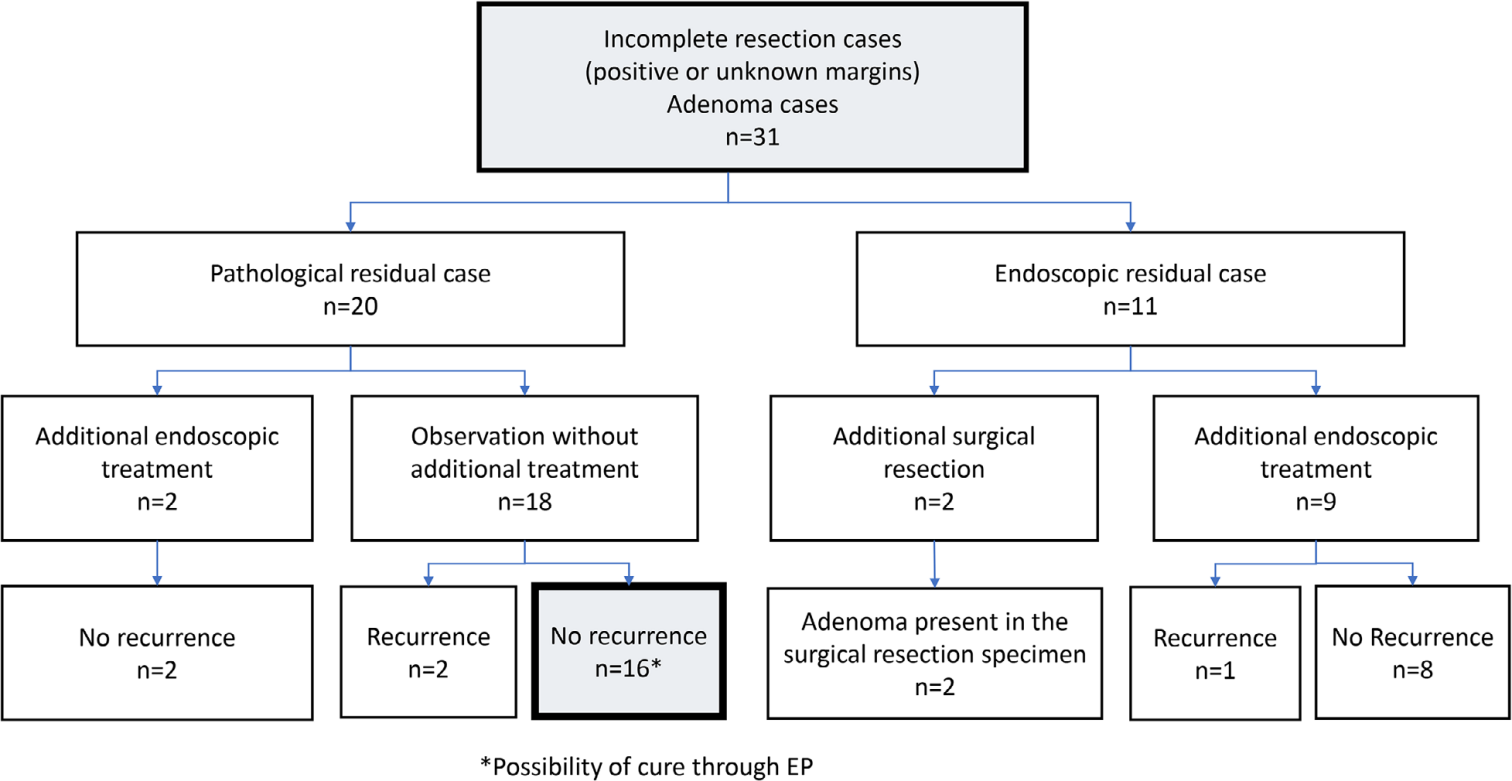
Clinical course of adenoma with positive or unknown endoscopic papillectomy (EP) margin. Among 31 cases of adenoma with positive or unknown pathological margins, 11 were classified as endoscopic residual adenomas, where residual adenomatous tissue was identified via endoscopic observation and biopsy following EP. The remaining 20 cases were categorized as pathological residual adenomas, in which no visible residual lesion was detected endoscopically after EP. EP, endoscopic papillectomy.

Recurrence occurred in two observed pathological residual cases and in one endoscopic residual case. All recurrences were controlled with repeat endoscopic treatment. After excluding four pathological residual cases with follow‐up shorter than one year, 16 cases were eligible for curative outcome analysis. No cases with endoscopic residual cases were excluded. The curative resection rate for EP was 75.0% (12/16) in pathological residual cases but 0% (0/11) in endoscopic residual cases (*p* < 0.001). Although three cases developed recurrence, all were successfully managed with repeat endoscopic treatment. The clinical course of adenoma pathology and endoscopic residual disease is detailed in Figure 3. Details of additional treatment cases and observation cases in Adenoma were shown in Table S5.

Cases showing positive/unknown or negative vertical margins are shown in Table S6, while cases where positive/unknown or negative horizontal margins are shown in Table S7.

### 3.4 Results of Additional Surgical Treatment

The outcomes of additional surgical treatment are summarized in Table 4. Among adenocarcinoma cases, five patients underwent pancreatoduodenectomy following EP. For adenoma cases, two patients underwent transduodenal papillectomy as an additional surgical intervention.

**TABLE 4:** Results of the additional surgical treatment.

	Adenocarcinoma (*n* = 5)	Adenoma (*n* = 2)
Surgical procedure (%)		
Pancreatoduodenectomy	5 (100)	0 (0)
Transduodenal papillectomy	0 (0)	2 (100)
Tumor in surgical resection specimen (%)		
Absence	4 (80.0)	0 (0)
Presence	1 (20.0)	2 (100)
Resection margin		
R0	−	2 (100)
R1	1 (20.0)	−
R2	−	−
Lymph node metastasis at surgical resection (%)	0 (0)	0 (0)
Operation time median (range), min	389 (281–504)	249 (225–273)
Blood loss median (range), mL	375 (227–400)	130 (96–165)
Hospital stay median (range), days	25 (17–27)	11 (8–14)
Re‐surgery rate (%)	1 (20.0)	0 (0)
Surgical morbidity (%)	0 (0)	0 (0)
Hospital mortality	0 (0)	0 (0)
Pancreatic fistula B/Cb	0 (0)	0 (0)
Postoperative bleeding	1 (20.0)	0 (0)
Ileus	1 (20.0)	0 (0)
Endocrine dysfunction	1 (20.0)	0 (0)
Exocrine dysfunction	1 (20.0)	0 (0)

Surgical adverse events included one case each of pancreatic fistula (B/Cb), ileus, exocrine dysfunction, and endocrine dysfunction. No perioperative mortality was observed.

## 4 Discussion

This study demonstrates that when endoscopic residual disease is identified after EP for duodenal ampullary tumors, additional treatment—either surgical or endoscopic—is essential, as tumor remnants are highly likely to persist and can progress to metastasis if left untreated. In contrast, cases with only pathological residual disease but no endoscopic evidence of residual tumor frequently showed no viable cancer on subsequent surgical specimens and did not experience recurrence during long‐term follow‐up, suggesting that EP alone may be curative in such situations. For adenomas, although recurrence can occur in cases managed conservatively, additional endoscopic therapy generally achieves good control. These results indicate that curative resection may have been achieved in a substantial proportion of pathological residual cases without endoscopic residual disease. Unlike endoscopic submucosal dissection or endoscopic mucosal resection, EP does not require local injection, allowing for closer contact between the resection margin and the tumor. Additionally, the thermal effect of cauterization may contribute to lesion eradication at the resection margins. Previous studies have suggested that the high incidence of positive or unknown margins in EP may result from artifacts caused by the resection technique [14, 15]. The thermal effect of EP may complicate pathological margin assessment, making it difficult to determine true residual disease. In previous reports, additional surgical resection in cases with positive or unknown pathological margins revealed no residual cancer in three out of four cases [3]. These findings suggest that a curative resection may still be obtained, even in cases where the pathological margin is positive or unknown.

Currently, no standardized strategy exists for additional treatment of residual adenocarcinoma after EP. When the final pathology of cases undergoing EP is situ carcinoma or mucosal lesions, the risk of lymph node metastasis is low [16, 17, 18]. However, additional surgical treatment for ampullary cancer is significantly more invasive than endoscopic treatment, and perioperative mortality rates are higher [19, 20, 21, 22]. In this study, no deaths occurred due to surgical adverse events among patients who underwent additional resection, although a case required reoperation for postoperative bleeding. Reports on non‐ampullary duodenal cancer suggest that endoscopic therapy provides a favorable prognosis for residual or recurrent mucosal lesions [23, 24, 25, 26]. Accordingly, the Japanese non‐ampullary duodenal cancer guidelines recommend that for non‐ampullary intraepithelial carcinoma (Tis) and mucosal (T1a) duodenal cancer, if follow‐up endoscopy confirms no residual disease, observation may be considered even when the horizontal margin is positive, rather than proceeding directly to surgical resection [27].

A similar strategy may also be appropriate for patients with duodenal papillary cancer. Therefore, it may be reasonable to carefully monitor cases with positive or unknown margins, provided that no endoscopic residual disease is detected. For residual or recurrent endoscopic lesions, if the tumor is limited to the mucosa, additional treatment may not necessarily require surgery. In such cases, local therapies, such as re‐EP, APC [3], or RFA [28, 29]. Conversely, if tumor invasion extends beyond the muscularis propria, the risk of lymph node metastasis increases [30]. Furthermore, cases with positive vascular invasion are associated with poor prognosis [31], making additional surgical resection the preferred approach.

Among the 11 cases of endoscopic residual adenoma, two were treated surgically, while the remaining nine underwent additional endoscopic therapy. A total of three cases of recurrence following adenoma EP were observed, but all were successfully controlled with endoscopic treatment. Previous studies have demonstrated that recurrent or residual ampullary adenomas can be effectively treated with endoscopic therapy, particularly using APC [3, 32]. These findings suggest that residual adenomas are likely to be well controlled with endoscopic management. Additionally, two cases of pathological residual disease without endoscopic residuals underwent additional endoscopic treatment. Studies have explored the role of RFA in reducing tumor recurrence [28, 29]. However, the efficacy of RFA in preventing residual and recurrent disease in pathological residual adenomas remains unknown, and its clinical utility requires further investigation.

This study has some limitations. It was a retrospective analysis with a small sample size, particularly in cases of residual adenocarcinoma. The limited number of cases restricts the statistical power of the findings, and further prospective studies with larger cohorts are necessary to validate these results.

In conclusion, although careful evaluation for possible residual carcinoma at the resection site is essential, in cases of adenocarcinoma with positive or unknown pathological margins without endoscopic residual disease following EP, observation may be carefully considered in selected cases with caution. However, endoscopic residual cases of adenocarcinoma require additional treatment to prevent progression. For adenomas, cases of pathological residual disease may also be monitored through follow‐up observation, whereas endoscopic residual cases should be considered for additional endoscopic treatment to achieve complete eradication.

## Author Contributions

Yusuke Kurita contributed to the study conception and design. Keiki Nagai and Noriki Kasuga performed data collection and analysis. Hiroki Uechi, Yuji Koyama, Shintaro Tsujikawa, Shota Matsumoto, Shigeki Tamura, Yu Honda, Shin Yagi, Takeshi Iizuka, Sho Hasegawa, and Kunihiro Hosono performed endoscopic treatments and collected data. Yuki Honma and Ryusei Matsuyama performed surgical treatments and collected data. Jotaro Harada conducted a pathology review and collected data. Kensuke Kubota, Yuji Fujita, Yusuke Sekino, Noritoshi Kobayashi, Masato Yoneda, Satoshi Fujii, Itaru Endo, and Atsushi Nakajima were responsible for manuscript drafting and critical revisions. All authors reviewed and approved the final version of the manuscript.

## Funding

The authors have nothing to report.

## Ethics Statement

**Approval of the research protocol by an Institutional Reviewer Board**: (F241200003).

## Consent

N/A. This study was conducted using an opt‐out form for retrospective research purposes.

## Conflicts of Interest

The authors declare no conflicts of interest.

## Supporting information




**Supplemental File**: deo270374‐sup‐0001‐TableS1‐S6.docx
