# Supplementary material for: Evaluation of Additional Treatment for Residual Cases After Endoscopic Papillectomy of Duodenal Ampullary Tumors
Source: DEN Open. 2026 Jul 1;7(1):e70374. doi: 10.1002/deo2.70374 (PMC13322654; doi:10.1002/deo2.70374)
Supplement: Supplementary file 1 — Supplemental File: deo270374‐sup‐0001‐TableS1‐S6.docx [file DEO2-7-e70374-s001.docx]

Supplementary Table 1. Detail of Additional Treatment Cases and Observation Cases in Adenocarcinoma cases

| Adenocarcinoma  Total (n=12) | Additional surgical resection (n = 5) | Observation  (n =7) | p |
| --- | --- | --- | --- |
| Age, median (range), years | 66 (53–80) | 75.0 (62–88) | 0.432 |
| Sex, male (%) | 3 (60.0) | 4 (57.1) | 1.000 |
| Status of residual  Pathological / Endoscopic | 4 (80.0) / 1 (20.0) | 6 (85.7) / 1 (14.3) | 1.000 |
| Tumor size, median (range), mm in EP | 15.0 (12–21) | 16.0 (14–20) | 0.639 |
| Mode of resection in EP (%)  En bloc/Piecemeal | 4 (80.0) / 1 (20.0) | 7 (100) / 0 (0) | 1.000 |
| Final pathological diagnosis of EP  Tis/T1a(M)/T1a(OD) | 4 / 0 / 1 | 3 / 2 / 2 | 0.332 |
| Histological grade (%)  Tub1/Tub2 | 5 (100) / 0 (0) | 5 (71.4) / 2 (28.6) | 0.470 |
| Histological subtype (%)  　Intestinal/Pancreatobiliary | 5 (100) / 0 (0) | 5 (71.4) / 2 (28.6) | 0.470 |
| Lymphovascular invasion (%) | 0 (0) | 0 (0) | NA |
| Resected margin of EP (-)/(+)/(x)  Horizontal (-)/(+)/(x)  Vertical (-)/(+)/(x) | 0/3/2  3/2/0  2/1/2 | 0/3/4  6/0/1  1/3/3 | 1.000  0.152  0.539 |
| Adenocarcinoma in additional surgical specimens (%)  Absence  Presence | 4 (80.0)  1 (20.0) | - | NA |
| Additional endoscopic treatment (%) | 0 (0) | 0 (0) | NA |
| Follow-up period after EP, month, mean (range) | 55.7 (18.4–86.6) | 23.0 (13.6–68.8) | 0.268 |
| Growth of primary tumor or recurrence (%) | 0 (0) | 1 (14.3) | 1.000 |
| Status of clinical follow-up (%)  Death | 0 (0) | 0 (0) | NA |
| Possibility of curative resection via EP (%) | 80.0 % (4/5) | 85.7% (6/7) | 1.000 |

EP, endoscopic papillectomy

Supplementary Table 2. Details of Positive and Unknown Margins in Adenocarcinoma Cases

| Adenocarcinoma  Total (n=12) | Margin of EP (+) (n = 6) | Margin of EP (x) (n = 6) | p |
| --- | --- | --- | --- |
| Status of residual  Pathological / Endoscopic | 5 (88.3) / 1(16.7) | 5 (88.3) / 1(16.7) | 1.000 |
| Tumor size, median (range), mm in EP | 15.0 (12–20) | 17.0 (10–21) | 0.485 |
| Mode of resection in EP (%)  En bloc/Piecemeal | 5 (88.3) / 1(16.7) | 6 (100) / 0 (0) | 0.296 |
| Final pathological diagnosis of EP  Tis/T1a(M)/T1a(OD) | 5 / 1 / 0 | 2 / 1 / 3 | 0.117 |
| Histological grade (%)  Tub1/Tub2 | 5 (88.3) / 1(16.7) | 5 (88.3) / 1(16.7) | 1.000 |
| Histological subtype (%)  　Intestinal/Pancreatobiliary | 5 (88.3) / 1(16.7) | 5 (88.3) / 1(16.7) | 1.000 |
| Lymphovascular invasion (%) | 0 (0) | 0 (0) | NA |
| Additional surgical resection (%) | 3 (50.0) | 2 (33.3) | 1.000 |
| Adenocarcinoma in additional surgical specimens (%)  Absence  Presence | 2 (33.3)  1 (16.7) | 2 (33.3)  - | 0.565 |
| Additional endoscopic treatment (%) | 0 (0) | 0 (0) | NA |
| Observation without additional treatment (%) | 3 (50.0) | 4 (66.7) | 1.000 |
| Follow-up period after EP, month, mean (range) | 30.6 (13.6–68.8) | 56.2 (21.0–86.6) | 0.394 |
| Growth of primary tumor or recurrence (%) | 0 (0) | 1 (16.7) | 1.000 |
| Status of clinical follow-up (%)  Death | 0 (0) | 0 (0) | NA |
| Possibility of curative resection via EP (%) | 88.3% (5/6) | 88.3% (5/6) | 1.000 |

EP, endoscopic papillectomy

Supplementary Table 3. Results based on findings from vertical margins of adenocarcinoma

| Adenocarcinoma  Total (n=12) | Vertical margin of EP (+)/(x) (n = 9) | Vertical margin of EP (-) (n =3) | p |
| --- | --- | --- | --- |
| Status of residual  Pathological / Endoscopic | 8 (88.9)/1(11.1) | 2 (66.7) / 1 (33.3) | 0.455 |
| Tumor size, median (range), mm in EP | 20.0 (12–20) | 16.0 (10–21) | 0.727 |
| Mode of resection in EP (%)  En bloc/Piecemeal | 9 (100)/0 (0) | 2 (66.7)/1 (33.3) | 1.000 |
| Final pathological diagnosis of EP  Tis/T1a(M)/T1a(OD) | 5/1/3 | 2/0/1 | 0.424 |
| Histological grade (%)  Tub1/Tub2 | 7 (77.8)/2 (22.2) | 3 (100)/0 (0) | 1.000 |
| Histological subtype (%)  　Intestinal/Pancreatobiliary | 7 (77.8)/2 (22.2) | 3 (100)/0 (0) | 1.000 |
| Lymphovascular invasion (%) | 0 (0) | 0 (0) | NA |
| Additional surgical resection (%) | 3 (33.3) | 2 (66.7) | 0.523 |
| Adenocarcinoma in additional surgical specimens (%)  Absence  Presence | 3 (33.3)  0 (0) | 1 (33.3)  1 (33.3) | 0.400 |
| Additional endoscopic treatment (%) | 0 (0) | 0 (0) | NA |
| Observation without additional treatment (%) | 6 (66.7) | 1 (33.3) | 0.180 |
| Follow-up period after EP, month, mean (range) | 58.1 (13.6–86.6) | 21.5 (18.4–38.2) | 0.209 |
| Growth of primary tumor or recurrence (%) | 1 (11.1) | 0 (0) | 1.000 |
| Status of clinical follow-up (%)  Death | 0 (0) | 0 (0) | NA |
| Possibility of curative resection via EP (%) | 88.9 % (8/9) | 66.7% (2/3) | 0.455 |

EP, endoscopic papillectomy

Supplementary Table 4. Results based on findings from horizontal margins of adenocarcinoma

| Adenocarcinoma  Total (n=12) | Horizontal margin of EP (+)/(x) n = 3 | Horizontal margin of EP (-) n = 9 | p |
| --- | --- | --- | --- |
| Status of residual  Pathological / Endoscopic | 2 (66.7) / 1 (33.3) | 8 (88.9)/1(11.1) | 0.455 |
| Tumor size, median (range), mm in EP | 14.0 (10–21) | 20.0 (12–20) | 0.727 |
| Mode of resection in EP (%)  En bloc/Piecemeal | 2 (66.7)/1 (33.3) | 9 (100)/0 (0) | 1.000 |
| Final pathological diagnosis of EP  Tis/T1a(M)/T1a(OD) | 2/0/1 | 5/1/3 | 0.424 |
| Histological grade (%)  Tub1/Tub2 | 3 (100)/0 (0) | 7 (77.8)/2 (22.2) | 1.000 |
| Histological subtype (%)  　Intestinal/Pancreatobiliary | 3 (100)/0 (0) | 7 (77.8)/2 (22.2) | 1.000 |
| Lymphovascular invasion (%) | 0 (0) | 0 (0) | NA |
| Additional surgical resection (%) | 2 (66.7) | 3 (33.3) | 0.523 |
| Adenocarcinoma in additional surgical specimens (%)  Absence  Presence | 1 (33.3)  1 (33.3) | 3 (33.3)  0 (0) | 0.400 |
| Additional endoscopic treatment (%) | 0 (0) | 0 (0) | NA |
| Observation without additional treatment (%) | 1 (33.3) | 6 (66.7) | 0.180 |
| Follow-up period after EP, month, mean (range) | 21.5 (18.4–38.2) | 58.1 (13.6–86.6) | 0.209 |
| Growth of primary tumor or recurrence (%) | 0 (0) | 1 (11.1) | 1.000 |
| Status of clinical follow-up (%)  Death | 0 (0) | 0 (0) | NA |
| Possibility of curative resection via EP (%) | 66.7% (2/3) | 88.9 % (8/9) | 0.455 |

EP, endoscopic papillectomy

Supplementary Table 5. Details of Additional Treatment Cases and Observation Cases in Adenoma cases

| Adenoma  Total (n=31) | Additional treatment (n = 13) | Observation  (n = 18) | p |
| --- | --- | --- | --- |
| Age, median (range), years | 63 (38–73) | 71 (59–83) | 0.008 |
| Sex, male (%) | 7 (53.8) | 11 (61.1) | 0.686 |
| Status of residual  Pathological / Endoscopic | 2 (15.4) / 11 (84.6) | 18 (100) / 0 (0) | < 0.001 |
| Tumor size (mm), median (range), in EP | 12 (5–20) | 12 (5–20) | 0.767 |
| Mode of resection of EP (%)  En bloc  Piecemeal | 10 (76.9)  3 (23.1) | 16 (88.9)  2 (11.1) | 0.625 |
| Resected margin of EP (-)/(+)/(x)  Horizontal (-)/(+)/(x)  Vertical (-)/(+)/(x) | 0/5/8  3/1/9  2/4/7 | 0/7/11  7/2/9  1/7/10 | 0.981  0.561  0.638 |
| Additional surgical resection (%) | 2 (15.4) | 0 (0) | 0.168 |
| Adenoma in additional surgical specimens (%)  Absence  Presence | -  2 (15.4) | -  - | NA |
| Additional endoscopic treatment (%)  Re-EP  APC  RFA  Details of treatment unknown | 11 (84.6)  5 (38.5)  4 (30.8)  1 (7.7)  1 (7.7) | 0 (0)  0 (0)  0 (0)  0 (0)  0 (0) | < 0.001 |
| Follow-up period after EP, months, mean (range) | 51.9 (3.4–151.9) | 24.4 (0.7–128.2) | 0.242 |
| Growth of primary tumor or recurrence (%) | 1 (7.7) | 2 (11.1) | 1.000 |
| Status of clinical follow-up　(%)  Death due to duodenal ampullary tumor  Death due to other causes | 0 (0)  1 (7.7) | 0 (0)  1 (5.6) | NA  1.000 |
| Possibility of curative resection via EP (%) | 0% (0/13) | 85.7 % (12/14) | < 0.001 |

APC, argon plasma coagulation; EP, endoscopic papillectomy; RFA, radiofrequency ablation

* 4 cases was excluded: Cases lacking at least 1 year of follow-up data after EP were excluded from the analysis of curative resection outcomes.

Supplementary Table 6. Results based on findings from vertical margins of adenoma

| Adenoma  Total (n=31) | Vertical margin of EP (+)/(x) (n = 28) | Vertical margin of EP (-) (n = 3) | p |
| --- | --- | --- | --- |
| Status of residual  Pathological / Endoscopic | 19 (67.9) / 9 (32.1) | 1 (33.3) / 2 (66.7) | 0.281 |
| Tumor size (mm), median (range), in EP | 12 (5–20) | 12 (11–13) | 0.925 |
| Mode of resection of EP (%)  En bloc  Piecemeal | 23 (82.1)  5 (17.9) | 3 (100)  0 (0) | 1.000 |
| Additional surgical resection (%) | 2 (7.1) | 0 (0) | 1.000 |
| Adenoma in additional surgical specimens (%)  Absence  Presence | -  2 (7.1) | -  - | NA |
| Additional endoscopic treatment (%)  Re-EP  APC  RFA  Details of treatment unknown | 9 (32.1)  4 (14.3)  3 (10.7)  1 (3.6)  1 (3.6) | 2 (66.7)  1 (33.3)  1 (33.3)  0 (0)  0 (0) | 0.782 |
| Observation without additional treatment (%) | 17 (60.7) | 1(33.3) | 0.558 |
| Follow-up period after EP, months, mean (range) | 24.4 (0.7–151.9) | 51.8. (39.6–55.2) | 0.349 |
| Growth of primary tumor or recurrence (%) | 2 (7.1) | 1 (33.3) | 0.271 |
| Status of clinical follow-up　(%)  Death due to duodenal ampullary tumor  Death due to other causes | 0 (0)  2 (7.1) | 0 (0)  0 (0) | NA  1.000 |
| Possibility of curative resection via EP (%) | 50.0% (12/24) | 0 % (0/3) | 0.231 |

APC, argon plasma coagulation; EP, endoscopic papillectomy; RFA, radiofrequency ablation

Supplementary Table 7. Results based on findings from horizontal margins of adenoma

| Adenoma  Total (n=31) | Horizontal margin of EP (+)/(x) (n =21) | Horizontal margin of EP (-) (n =10) | p |
| --- | --- | --- | --- |
| Status of residual  Pathological / Endoscopic | 12 (57.1) / 9 (42.9) | 8 (80.0) / 2 (20.0) | 0.262 |
| Tumor size (mm), median (range), in EP | 12 (5–20) | 12.5 (7–15) | 0.441 |
| Mode of resection of EP (%)  En bloc  Piecemeal | 16 (76.2)  5 (23.8) | 10 (100)  0 (0) | 0.147 |
| Additional surgical resection (%) | 2 (9.5) | 0 (0) | 1.000 |
| Adenoma in additional surgical specimens (%)  Absence  Presence | -  2 (9.5) | -  - | NA |
| Additional endoscopic treatment (%)  Re-EP  APC  RFA  Details of treatment unknown | 8 (38.1)  4 (19.0)  3 (14.3)  0 (0)  1 (4.8) | 3 (30.0)  1 (10.0)  1 (10.0)  1 (10.0)  0 (0) | 0.503 |
| Observation without additional treatment (%) | 11 (52.4) | 7 (70.0) | 0.452 |
| Follow-up period after EP, months, mean (range) | 24.5 (2.2–151.9) | 32.8 (0.7–128.2) | 0.603 |
| Growth of primary tumor or recurrence (%) | 3 (14.3) | 0 (0) | 0.553 |
| Status of clinical follow-up　(%)  Death due to duodenal ampullary tumor  Death due to other causes | 0 (0)  2 (9.5) | 0 (0)  0 (0) | NA  1.000 |
| Possibility of curative resection via EP (%) | 40.0 % (8/20) | 57.1 % (4/7) | 0.662 |

APC, argon plasma coagulation; EP, endoscopic papillectomy; RFA, radiofrequency ablation
